# Nonventilatory factors affecting noninvasive mechanical ventilation success in hypercapnic critical care patients

**DOI:** 10.1186/cc13453

**Published:** 2014-03-17

**Authors:** S Demir, Y Aldag, M Aydogdu, G Gürsel

**Affiliations:** 1 Gazi University Medical Faculty, Ankara, Turkey

## Introduction

The aim was to determine the nonventilatory factors that affect the noninvasive mechanical ventilation (NIMV) success in patients with hypercapnic respiratory failure.

## Methods

A total of 41 patients were included in this prospective cohort study, who were followed for at least 96 hours in the ICU between January 2010 and November 2012 with the diagnosis of hypercapnic respiratory failure. Patients with ≥10 mmHg decrease in PaCO_2 _in the first 72 hours were accepted as successful (Group 1) and those without this decrease were accepted as unsuccessful (Group 2). Among the patients with similar NIMV application characteristics, the effect of age, APACHE II score, infection, bronchospasm (daily respiratory function tests were performed with portable spirometry), heart failure, thyroid dysfunction and physiologic dead space ventilation (VD/VT) on success were evaluated. In statistical analysis, t test, Mann-Whitney U test and regression analysis were used.

## Results

Among the 41 patients, 28 (68%) were classified as Group 1 and 13 (32%) as Group 2. No differences were identified among the ventilatory parameters of the two groups (*P *> 0.05). Patients in Group 1 were younger, had higher admission PaCO_2 _levels, higher free T3 levels and ejection fraction in echocardiography (*P *< 0.05). VD/VT values of Group 1 measured at admission and on the second and third days of NIMV were lower than Group 2 (*P *< 0.05). Similarly, they had lower FEV1 percent predicted values in the stable period and on the first and second days of NIMV (*P *< 0.05). FEV1/FVC was lower in Group 1 when measured in the stable period and on the third and fourth days of NIMV. In Group 2, CRP values measured during the first day (*P *= 0.014) and third day (*P *= 0.031) of NIMV were identified as higher. In multivariate regression analysis, admission PaCO_2 _(OR: 1.59, 95% CI: 1.1 to 2.3, *P *= 0.014), free T3 (OR: 12, 95% CI: 1.51 to 101, *P *= 0.019), and VD/VT (OR: 1.23, 95% CI: 1.01 to 1.52, *P *= 0.048) were identified as independent risk factors affecting NIMV success.

## Conclusion

The results of this study revealed that among the NIMV nonresponsive hypercapnic patients, nonventilatory factors such as thyroid dysfunction and increased dead space ventilation should be considered.

